# Effect of Material Anisotropy on the Mechanical Response of Automotive Steel under High Strain Rates

**DOI:** 10.3390/ma15020669

**Published:** 2022-01-17

**Authors:** Sheng Yin, Yi Xue, Haotian Cui, Xinhua Pei, Chundong Hu, Yangxin Wang, Qingchao Tian

**Affiliations:** 1 Technology Institute, Meishan Iron & Steel Co., Ltd., Nanjing 210000, China; yinsheng@baosteel.com (S.Y.); peixinhua@baosteel.com (X.P.); 2 State Key Laboratory of Advanced Special Steel, Shanghai University, Shanghai 200434, China; xueyi18@shu.edu.cn (Y.X.); cuihaotian@shu.edu.cn (H.C.); huchundong@shu.edu.cn (C.H.); wangyangxin@shu.edu.cn (Y.W.)

**Keywords:** dynamic deformation behavior, Cowper-Symonds model, texture, Hall–Petch relationship, microstructure

## Abstract

A constitutive model for automobile steel with high elongation needs to be established to predict the dynamic deformation behavior under hydroforming applications. In order to clarify the confusing discrepancy in the essential parameters of the classical Cowper-Symonds (C-S) model, a series of automobile structural steels have been employed to investigate the strain rate response by conducting tensile dynamic deformation. Metallographic microscopy and orientation distribution functions were used to characterize the microstructure and texture components of the steels. The microstructure observation discloses that the matrix of all steels is mainly of ferrite and the texture constituent provides a framework for steel to withstand external deformation. The C-S model can be applied to simulate the dynamic deformation with satisfied expectations. It is concluded that the essential parameters *D* and *p* in the model show a specific relationship with the steel grade, and the parameter *D* is proportional to the steel grade and related to material anisotropy, while the parameter *p* is inversely proportional to the steel grade and has close links with the grain boundary characteristics.

## 1. Introduction

The mechanical behavior of automotive structural steels ensures the safety of automobile components during the occurrence of extreme collisions by the absorption of impact energy; therefore, the dependent variables of not only the quasi-static deformation but also the dynamic response to collision must be taken into account in order to predict the failure process under varied strain rate conditions [1,2]. A high-speed servohydraulic testing machine and modified Hopkinson bar system can be employed to record the mechanical behavior of materials over a wide range of strain rates [3,4]. Furthermore, researchers have designed different Hopkinson-like rods or employed resistance strain gauges to reduce the influence of stress fluctuation at high strain rates, and these attempts can reduce the fluctuation amplitude to a certain degree [5,6,7,8,9]; standard testing method has been established to obtain valid stress–strain data in the dynamic test with the consideration of stress equilibrium to avoid system ringing [10].

Since steel structure is very sensitive to the change of strain rate, diverse models have been established to predict the dynamic deformation behavior of structural steels [11,12,13,14,15]. The Johnson-Cook (J-C) model defines the constitutive relation of the effective yield stress with strain, strain rate and temperature [11]. In order to obtain an accurate rate-prediction model, Chen et al. [12,13] studied the mechanical behavior of Q420 and Q345 steels with strain rates from 0.001 s^−1^ to 288 s^−1^ and 0.001 s^−1^ to 330 s^−1^, respectively. Compared with the J-C models, a modified Hollomon-Voce model is proposed to predict the rate-dependent response. According to the dynamic response of S235, S690QL and S960QL, Alabi et al. [14,15] proposed the strain hardening exponent and strain sensitivity to explain the mechanical performance of these materials even in the presence of flaws. Nevertheless, the classical Cowper-Symonds (C-S) constitutive model has been widely applied to describe the performance of materials under dynamic deformation situations with good reliability [16,17].

Automobile steel plate with higher total elongation and moderate strength is required for some hydroforming applications [18,19], in which the deformation rate of different parts varies. Therefore, a constitutive model for such steels needs to be established to predict the dynamic deformation behavior under hydroforming conditions. However, confusing significant discrepancy has been found in the essential parameters *D* and *p* of the C-S model after conducting dynamic deformation of the steels in comparison with former research [20,21,22,23,24], and the influence factors seems to be in close relation with the material microstructural character, in which limited research studies have mentioned before. Whereas this paper firstly builds the C-S model of a series of structural steels by using a Zwick/Roell HTM5020 testing machine to minute the dynamic response, and then discloses the inherent regularity of the observed microstructure to the parameters of the C-S model.

## 2. Experimental

All test steels were prepared subsequently by vacuum melting, casting and hot rolling. The cast ingots were reheated to 1210–1235 °C, followed by hot-rolling to the thickness of about 2 mm with the finish temperature (FT) of 840 °C and the coiling temperature (CT) of about 550–570 °C. The chemical composition and rolling temperature of the steels are listed in Table 1; it can be seen that the steel sheets were characterized by low carbon content of 0.07 wt.% and were prepared from the viewpoint of economical use of alloying elements by gradually increasing the contents of Nb and Ti for heightened steel grade matching with controlled rolling and controlled cooling process. The varied steel grades in Table 1 are expected to help understand the effect of orientation on the mechanical response of such type of automotive steels.

**Figure 1:**
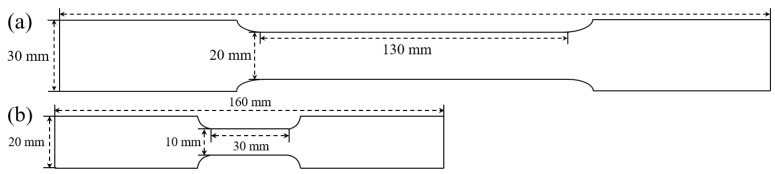
The geometry of (**a**) quasi-static and (**b**) dynamic tensile specimens.

All tensile specimens used in this study were spark cut from the prepared steel sheet along the rolling and the transvers directions, respectively, and the geometry of quasi-static (EN ISO 6892-1 2009) and dynamic specimens (ISO 26203-2 2011) are shown in Figure 1, guaranteeing that the round-trip number of the stress wave is higher than 10 times to assure the validity of the stress–strain data [5,20,21,22]. It is noted that the different sampling norms are considered from engineering practice, and the elongation of small-size specimen is slightly higher than that of the large-size one, while yield strength and tensile strength are identical.

A standard tensile test was conducted under a strain rate of 0.001 s^−1^ using a universal electromechanical testing machine (ZwickRoell, Ulm, Germany), and dynamic tests were carried out at 1, 2, 3, 4, 5, 10 and 18 m/s tensile speed corresponding to the nominal strain rates of 33, 66, 100, 133, 167, 333 and 600/s, respectively.

Before the dynamic test, the surface of the deformation area of the samples was sprayed with a white background and then decorated by random black spots to be sure that the speckle patterns were of appropriate gray distribution for recording. That is, the subset size is a 20 × 20 pixel square, the distance between two elements is 12 pixels. According to the image resolution of 40–45 pixels/mm, an approximate 0.5–0.6 mm element size which is equivalent to two-times the subset distance. The tensile deformation was instantaneously recorded by a high-speed camera (Vision Resesarc Inc., Wayne, NJ, USA) [25]. The load data recorded by the weight sensor (ZwickRoell, Ulm, Germany) is used to calculate the engineering stress. The sampling frequency of the weighing sensor matches that of the high-speed camera (60,000 frames per second) to maintain synchronization. The engineering stress–strain curves under each strain rate are obtained by computer software processing.

The microstructures were observed by metallographic microscopy (OM) (Leica, Solms, Germany) at the 1/4-thickness position of the hot rolled sheets along the longitudinal section. The specimens were firstly planning machined to the target plane and then etched after mechanical polishing by using a 4% nital solution. Samples for texture measurements were spark cut with the dimensions of 20 × 10 mm, and a Bruker™ D8 Discover system (Bruker, Karlsruhe, Germany) with a CuKa target were employed under a scanning speed of 8°/min and operating at voltages of 40 kV and 40 mA current.

## 3. Results and Discussion

### 3.1. Microstructure Characteristics

**Figure 2:**
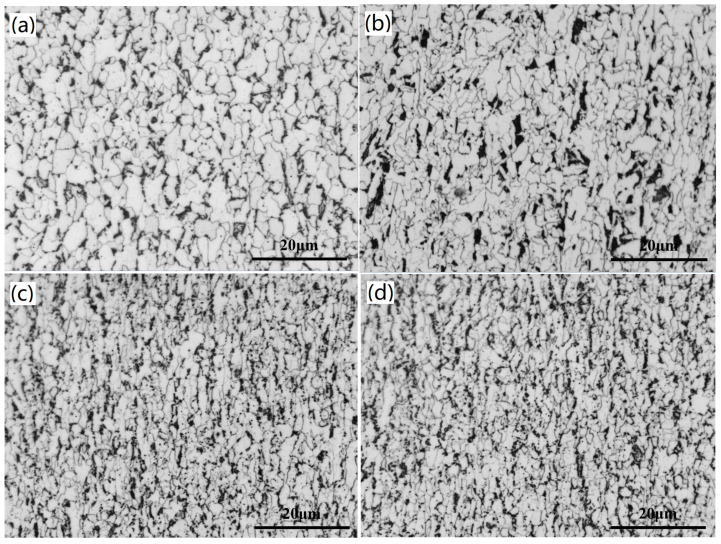
Microstructures of the hot rolled sheets along the rolling direction (**a**) Q380, (**b**) Q420, (**c**) Q460 and (**d**) Q500.

Figure 2 shows the microstructures at the 1/4-thickness position along the longitudinal section. All the microstructures exhibit a slab-like bright white phase and black areas. The white areas are of ferrite while the black ones are of pearlite in Q380 and Q420 steels. As for Q460 and Q500, the black areas consist of pearlite and a small quantity of bainite. The ferrite grain of Q380 is more multi-scaled with an average size of 4.59 μm; with the increase of steel grade, the grain becomes finer and finer. The grain sizes of Q420, Q460 and Q500 are about 3.24 μm, 2.82 μm and 2.70 μm, respectively.

According to the Hall–Petch equation [23,24]:

(1) $$\sigma =\sigma_{0}+k_{\mathrm{HP}}d^{-0.5}$$

where σ is the yield stress, $\sigma_{0}$ is constant and *d* is the grain size, $k_{HP}$ is the Hall–Petch coefficient related to the grain boundary strengthening effect.

According to the measured yield stress and grain size in this paper and that in the Refs. [18,19], the $\sigma_{0}$ and $k_{HP}$ parameters for different steels can be calculated as listed in Table 2, in which can be seen that the $\sigma_{0}$ and $k_{HP}$ linearly increase with the rise in steel grade.

It is easy to understand that the fine grain can more efficiently hinder the dislocation movement and thus owe a better strengthening effect. It has been disclosed that $k_{HP}$ depends on the steel composition [26,27]. Nevertheless, the microstructure distribution, phase constitution and grain boundary characteristic (fraction and distribution of defects and grain boundary misorientation) also contribute to the $k_{HP}$. It is safe to say that $k_{HP}$ corresponds to the key microstructural parameter on mechanical property of steels.

**Figure 3:**
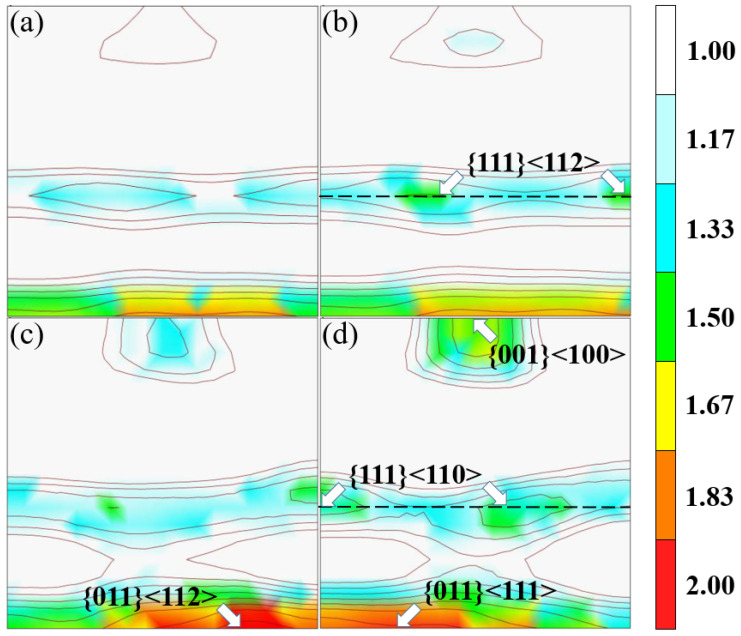
The texture distribution of the hot rolled sheets (**a**) Q380, (**b**) Q420, (**c**) Q460 and (**d**) Q500.

Figure 3 shows the texture components in the $\varphi_{2}$ = 45° sections of Euler space, where the variation regularity can be clearly identified. The texture constituent of the four steels is similar, and mainly consists of the {011}<111>, {001}<100> and the {111} fiber texture with a maximum intensity of about 2.00. With the increase of steel grade, the intensity of {001}<100> cube texture increases gradually, as well as that of the {011} texture. All steels possess the {111} γ fiber, where {111}<112> texture is obvious for steels Q380, Q420 and Q460, while {111}<110> texture is stronger for Q500. In comparison with the lower steel grades, Q460 and Q500 have stronger {011}<112> and {011}<111>, respectively [28].

### 3.2. Tensile Behavior along Rolling Direction

#### 3.2.1. Characteristic of Stress–Strain Curves

**Figure 4:**
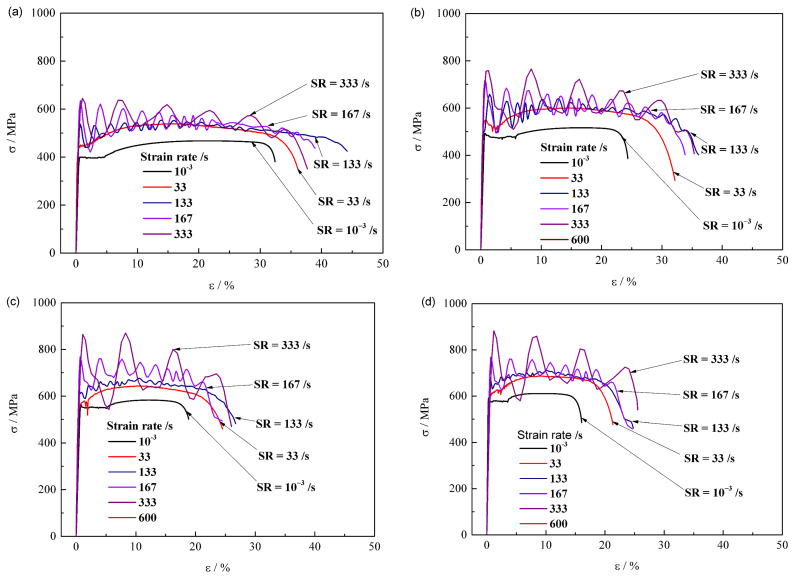
Stress–strain curves of longitudinal (**a**) Q380, (**b**) Q420, (**c**) Q460 and (**d**) Q500 at different strain rates.

Figure 4 represents the stress–strain curves of the steels along a rolling direction under different strain rates from 10^−3^ to 600 s^−^^1^. The standard test gives a typical engineering stress–strain curve with an obvious yield plateau. As the strain rate ($\dot{\varepsilon }$) increases, the plastic stage of stress–strain curve shows an overall upward trend of uplift, and appears to fluctuate in a periodic decay mode when the loading strain rate reaches higher than 100 s^−1^. With the increase of steel grade from Q380 (Figure 4a), to Q420 (Figure 4b), to Q460 (Figure 4c), and then to Q500 (Figure 4d), the variation of stress–strain curves exhibits a similar tendency, except the decrease in total elongation.

**Figure 5:**
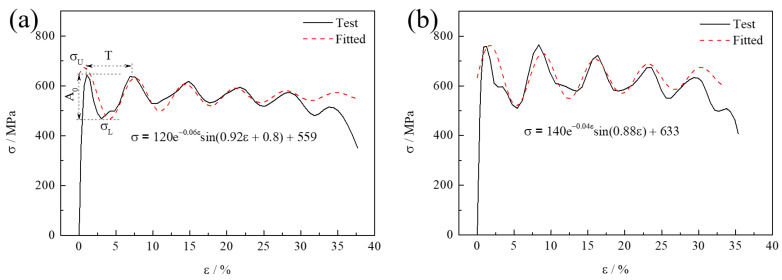
The fluctuation characteristic of stress-strain curves (**a**) Q380 and (**b**) Q420 steels at strain rate of 333 s^−1^, where the first appeared maximum stress is assigned as $\sigma_{U}$.

Figure 5 shows the characteristic of fluctuation, of which the stress–strain curve can be approximately fitted as a sine attenuation function ($\sigma =A_{0}e^{-\xi \varepsilon }\sin\left(\omega \varepsilon +\phi \right)\;+\;C_{0}$), where $C_{0}$ is the constant, and the distance between the first two crests and the distance between the first crest and the trough is taken as the cycle (*T*) and the amplitude ($A_{0}$), as shown in Figure 5a. In the high speed dynamic tensile test, the stress signal oscillation will inevitably occur owing to the increase of loading rate, and Alabi believed that it was the stress wave generated by the imbalance between internal friction and external force at high strains that caused the loading signal to be noisy [5,6,7,14,26,27,28,29]. Anyway, we assign the first appeared maximum stress $\sigma_{U}$ as upper yield stress to consider the strain-rate response of all specimens.

#### 3.2.2. Strain Rate Response

**Figure 6:**
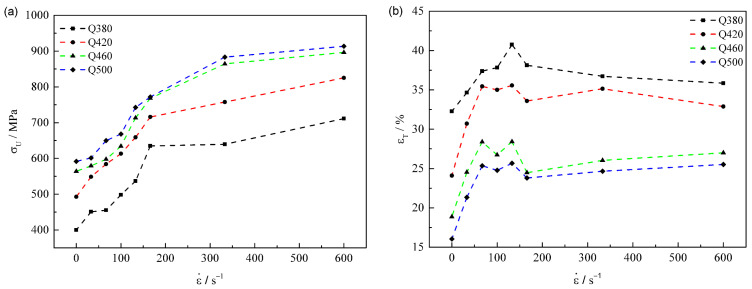
Dynamic response of (**a**) the upper yield stress ($\sigma_{U}$) and (**b**) the total elongation dependence of strain rate for different steels.

Figure 6 represents the dynamic response of the defined *σ_U_* and the total elongation of different steels. It can be seen in Figure 6a that the $\sigma_{U}$ value gradually increases to saturation with the increase of strain rate, while the $\sigma_{U}$ curve lifts up with the increase of steel grade (Q380: from 400 to 711 MPa, Q420: from 492 to 825 MPa, Q460: from 563 to 896 MPa and Q500: from 591 to 913 MPa). The variation tendency of $\varepsilon_{T}$ exhibits obvious discrepancy, as shown Figure 6b, the overall *ε_T_* curve shifts down with the increase of steel grade. With the increase of $\dot{\varepsilon }$, the $\varepsilon_{T}$ value first rapidly increases and then tends to a maximum value when $\dot{\varepsilon }$ reaches about 100 s^−1^ and then remains stabilized afterward. The stabilization $\varepsilon_{T}$ is about 37.8% for Q380, 34.7% for Q420, 25.8% for Q460 and 24.6% for Q500, respectively.

Then, the C-S constitutive model is introduced to further describe the above phenomenon. The dynamic increase factor (DIF_*y*_) was defined as the ratio of the yield stress at each intermediate strain rate ($\sigma_{i}$) to the yield stress at the quasi-static strain rate ($\sigma_{q}$):

(2) $${\mathrm{DIF}}_{y}=1+{\left(\frac{\dot{\varepsilon }}{D}\right)}^{\frac{1}{p}}$$

where *D* and *p* are the coefficient in the relation of stress with strain rate.

**Figure 7:**
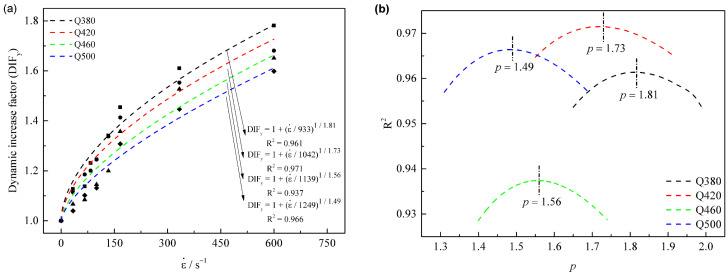
The C-S model fitting curves of Q380, Q420, Q460 and Q500 steels, (**a**) fitting results, (**b**) determination method for the *p* solution.

Figure 7 shows the longitudinal dynamic increase factor dependence of strain rate. Using the C-S model fitting (Figure 7a), the four steels can be well fitted and are described as $\mathrm{DIF}=1+{\left(\dot{\varepsilon }/933\right)}^{1/1.81}$ for Q380, $\mathrm{DIF}=1+{\left(\dot{\varepsilon }/1042\right)}^{1/1.73}$ for Q420, $\mathrm{DIF}=1+{\left(\dot{\varepsilon }/1139\right)}^{1/1.56}$ for Q460 and $\mathrm{DIF}=1+{\left(\dot{\varepsilon }/1249\right)}^{1/1.49}$ for Q500 with satisfied accuracy, respectively. It is noted that in the fitting process, the *p* value is continuously adjusted to seek for the best goodness-of-fit, and the highest R^2^ is determined to correspond to the right regression coefficients, as shown in the Figure 7b.

#### 3.2.3. Parameters Dependence

**Figure 8:**
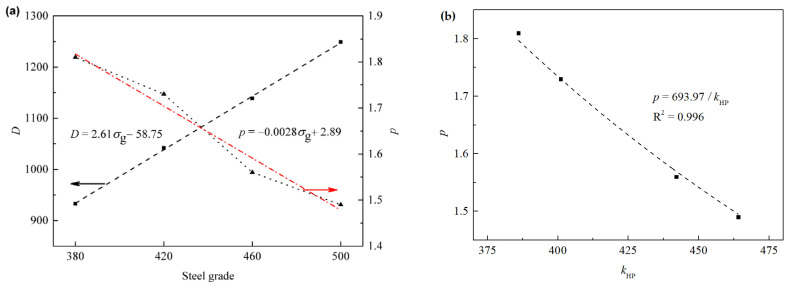
The variation of essential parameters in C-S model, (**a**) *D* and *p* dependence of steel grade $\sigma_{g}$ where the two arrows are pointed to two *Y*-axes of *D* and *p*, respectively, and (**b**) the *p* dependence of the Hall-Petch coefficient $k_{HP}$.

The graphs for the linear regression analysis based on the above data depending on steel grade are shown Figure 8a; the essential parameter *D* is proportional while *p* is inversely proportional to the steel grade, showing an amazingly strong contrast.

By comparing the parameter *p* in C-S model, we can see that there is a certain relationship $p=693.97/k_{HP}$ between them, as shown in Figure 8b.

Since the Hall–Petch equation is very essential for materials, and the $k_{HP}$ is easily available in the literatures, the C-S model can be replaced as:

(3) $${\mathrm{DIF}}_{y}=1+{\left(\frac{\dot{\varepsilon }}{D}\right)}^{\frac{K_{HP}}{673.79}}$$

and then the relationship between the dynamic growth factor of material and the influence coefficient of grain boundary on deformation is established, which shows that the dynamic growth factor may be related to the adhesion between grains and the coordination of grain deformation. The substitutive C-S model can be employed to estimate the dynamic response of a material according to the Hall–Patch coefficient.

### 3.3. Anisotropy on Strain Rate Response

Considering the anisotropy of the material, a tensile test was conducted using specimens prepared along the steel transverse direction to compare the dynamic tensile behavior with that along the longitudinal rolling direction.

**Figure 9:**
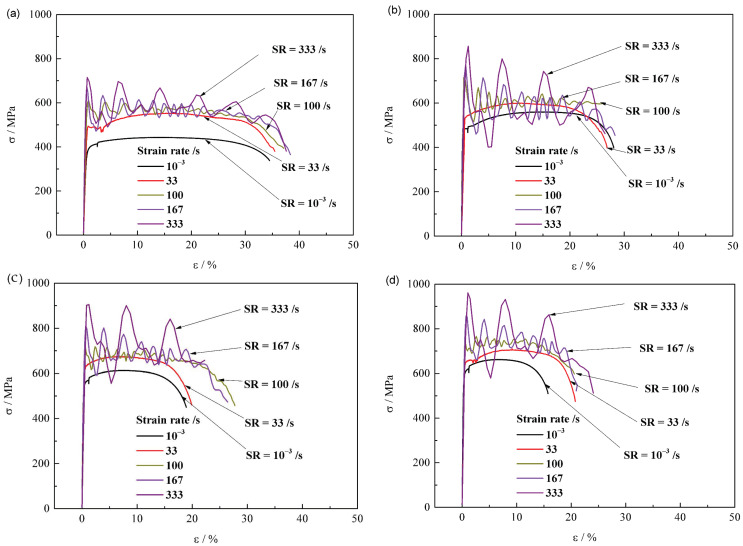
Stress-strain curves along transverse direction for specimens (**a**) Q380, (**b**) Q420, (**c**) Q460 and (**d**) Q500 at different strain rates.

Figure 9 shows the stress–strain curves of specimens along a transverse direction at different strain rates from 0.001 s^−1^ to 333 s^−1^. As for the same steel, the total elongation decreases for the specimen along transverse direction, which is different with that along the rolling direction. Similar to that along the longitudinal direction, with the increase of strain rate, the variation tendency of stress–strain curve is similar.

**Figure 10:**
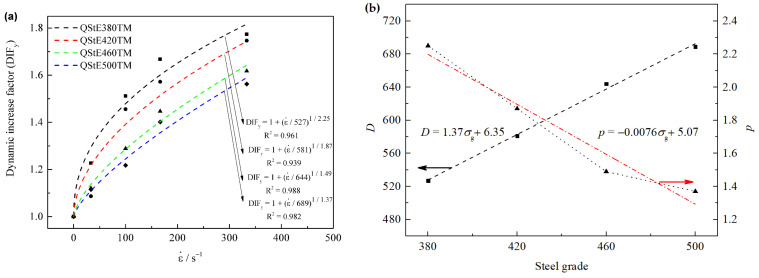
The C-S model fitting curves for steels along transvers direction, (**a**) fitting results, (**b**) *D* and *p* dependence of steel grade $\sigma_{g}$.

Figure 10 shows the transverse dynamic increase factor dependence of strain rate. Using the C-S model fitting (Figure 10a), the four steels can be well fitted with satisfied accuracy and are described as ${\mathrm{DIF}}_{y}=1+{\left(\dot{\varepsilon }/527\right)}^{1/2.25}$ for Q380, ${\mathrm{DIF}}_{y}=1+{\left(\dot{\varepsilon }/581\right)}^{1/1.87}$ for Q420, ${\mathrm{DIF}}_{y}=1+{\left(\dot{\varepsilon }/644\right)}^{1/1.49}$ for Q460 and ${\mathrm{DIF}}_{y}=1+{\left(\dot{\varepsilon }/689\right)}^{1/1.37}$ for Q500, respectively.

Based on the linear regression analysis of the above data (Figure 10b), the model parameter *D* is proportionate to the steel grade, while *p* is inversely proportionate to the steel grade.

### 3.4. Factors on Dynamic Deformation

The C-S model has been employed to simulate the dynamic response of different steels, as listed in Table 3 [12,13,14,17,30]. There exists significant difference in the characteristic parameters *D* and *p*, in particular, a vast gap can be found between the *D* values of different steels. It can be seen in Table 3 that all the steels are of low carbon type, and as for S355 steel, the metallurgical microstructure is of well-developed ferrite and pearlite [17]. Therefore, it is inferred that factors, such as material composition, microstructure and crystallographic texture determine the *D* and *p* values of materials.

The matrix of all the tested steels here is mainly of ferrite, and the comparison of tensile test along two different directions discloses that the main parameters of the C-S model exhibit a specific relationship with the microstructure, i.e., the parameter *D* is related to anisotropy while *p* has close links with the grain boundary characteristics.

In the process of plastic deformation, the stresses on the crystals of different orientations are not consistent owing to the anisotropy of crystal. The crystal grains do not deform at the same time, and their slip system orientation and slip direction are also different. As we all know, the {011} crystal plane of ferrite steel is densely packed, and it is the main slip plane of the bcc crystals, while the <111> direction is its main sliding direction. Therefore, the stronger {011} texture is beneficial to the dislocation slip, and thus is helpful to improve the ductility of the material. In contrast, the Burgers vector a<100> is a kind of immobile dislocation, and the texture of {001} is harmful to deformation at most cases. It has been pointed out that the {111} fiber texture is good for material forming but has little effect on improving the elongation of the material [19]. Therefore, there exists a competitive mechanism on plasticity between the textures {011} and {001}, and obviously the effect of {001} texture prevails for the steels in this study, and the increase in the intensity of {001}<100> cube texture leads to the decrease of total elongation with the increase of steel grade (Figure 6b). Additionally, it can be seen that the texture constituent provides a framework for steel to withstand external deformation.

The plastic deformation requires a certain amount of time for the movement of crystal dislocations, for the rotation of the slip plane from the unfavorable to the favorable orientation and the rotation between individual crystals. As large strain rate applies, only elastic deformation of the movement of the atoms away from their equilibrium position occurs owing to the insufficient duration for deformation, resulting in a persistent increase of yield stress with the increase of strain rate [31].

The change of elongation under high strain rate conditions might be related to the combined effect of thermal activation of dislocation movement and suppression of necking. Since plastic strain is mainly carried out by the slip of dislocations, on the one hand, high strain rate constrains the process of thermal activation of dislocations, dislocation multiplication should be dominant to increase the uniform elongation; on the other hand, high strain rate may harden the local constriction zone and expand the subsequent deformation to adjacent area; therefore, the ductility of material increases with the increase of strain rate.

## 4. Conclusions

A series of Q380, Q420, Q460 and Q500 steels have been prepared to investigate their dynamic deformation behavior. The main results are concluded as follows.

(1) The microstructures for Q380 and Q420 steels consist of ferrite and pearlite, while that for Q460 and Q500 consist of ferrite, pearlite and a small quantity of bainite. All steels mainly consist of the {011}<111>, {001}<100> and the {111} fiber texture with a maximum intensity of about 2.00. The ferrite texture constituent provides a framework for steel to withstand external deformation, and the increase in the intensity of {001}<100> cube texture leads to the decrease of total elongation with the increase of steel grade;
(2) Specimens for all the steels along both the rolling and the transverse directions show similar tensile behavior. As the strain rate increases, the stress–strain curve fluctuates in a periodic decay mode. The upper-yield stress increases with the increase of strain rate from 10^−3^ to 600 s^−1^, meanwhile, the total elongation first rapidly increases and then tends to be stabilized at certain values. The C-S model can be employed to well fit the strain rate response of the four steels both along transverse and rolling directions;
(3) Under the specific experiment conditions, the main parameters of the C-S model exhibit a certain relationship with the microstructure, the parameter *D* is proportional to the steel grade and related to material anisotropy, while *p* is inversely proportional to the steel grade and has close links with the grain boundary characteristics. The C-S model can be considered to estimate the dynamic response of a material according to the Hall–Patch coefficient.

## Figures and Tables

**Table 1:** Chemical composition and rolling temperature of the prepared steels.

Steel Grade	Chemical Composition/wt. %				Roll Temperature/°C	
	C	Si + Mn + Al	Nb	Ti	FT	CT
Q380	0.07	≤1.0	0.015	0.013	840	570
Q420	0.07	≤1.0	0.03	0.013	840	570
Q460	0.07	≤1.5	0.04	0.02	840	560
Q500	0.07	≤1.5	0.05	0.02	840	550

**Table 2:** Calculated parameters of Hall–Petch relationship for different steels.

Steel Type	Yield Stress/MPa	Grain Size/μm	*σ*_0_/MPa	$k_{HP}/\mathrm{MPa}\times \mu m^{0.5}$
Q380	429	4.59	249	386
	407 [18]	5.96 [18]		
Q420	493	3.24	271	401
	455 [18]	4.74 [18]		
Q460	556	2.82	293	442
	565 [19]	2.64 [19]		
Q500	592	2.70	310	464
	537 [18]	4.17 [18]		

**Table 3:** Chemical composition and the C-S model parameters of different steels.

Steels	Chemical Composition								The C-S Modeling	
	C	Si	Mn	P	S	Ni	V	Nb	*D*	*p*
Q420 [12]	0.19	0.24	1.35	0.021	0.006				22305	3.9
Q345 [13]	0.16	0.19	1.28	0.025	0.011				3781	4.33
S690 [14]	0.14	0.29	1.19	0.008	<0.002	0.084	0.031	0.016	2.06 × 10^7^	5.18
S960 [14]	0.16	0.21	1.39	0.008	<0.002	0.077	0.021	0.013	4.90 × 10^7^	4.59
S355 [17]	0.22	0.55	1.60	0.025	0.025				4945	2.69
Q235 [30]	0.22	0.35	1.4	0.045	0.05				306	2.75

Note: the parameters for S690 and S960 are deduced from the original data in Ref. [14].

## Data Availability

The data used to support the findings of this study are included within the article, additional data are available from the corresponding author upon request.
